# Using personalised brain stimulation to modulate social cognition in adults with autism-spectrum-disorder: protocol for a randomised single-blind rTMS study

**DOI:** 10.1186/s12888-025-06719-1

**Published:** 2025-03-25

**Authors:** Katia Ourania Brouzou, Daniel Kamp, Lukas Hensel, Jana Lüdtke, Juha M. Lahnakoski, Juergen Dukart, Nace Mikus, Christoph Mathys, Simon B. Eickhoff, Leonhard Schilbach

**Affiliations:** 1 Department of Psychiatry and Psychotherapy, Medical Faculty, Heinrich Heine University Düsseldorf, Düsseldorf, Germany; 2 Department of General Psychiatry 2, LVR-Klinikum Düsseldorf, Düsseldorf, Germany; 3 Institute of Systems Neuroscience, Heinrich Heine University Düsseldorf, Düsseldorf, Germany; 4 Institute of Neuroscience and Medicine, Brain and Behaviour (INM-7), Research Center Juelich, Juelich, Germany; 5 Interacting Minds Centre, Aarhus University, Aarhus, Denmark; 6 Translational Neuromodeling Unit (TNU), Institute for Biomedical Engineering, University of Zurich and ETH Zurich, Zurich, Switzerland; 7 Department of Psychiatry and Psychotherapy, Medical Faculty, Ludwig Maximilians University Munich, Munich, Germany

**Keywords:** Autism spectrum disorder, Transcranial magnetic stimulation, Personalized neuronavigation, Social cognition

## Abstract

**Background:**

Autism spectrum disorder (ASD) is a neurodevelopmental disorder characterized by impairments of social interaction and communication as well as repetitive, stereotyped behaviour. Previous research indicates that ASD without intellectual impairment is associated with underactivity and reduced functional connectivity of the brain’s mentalizing pathway, to which the right temporo-parietal junction (rTPJ) serves as an important entry point and hub. In this study, we aim to utilize functional magnetic resonance imaging (fMRI) to localize activation maxima in the rTPJ and other regions involved in social cognition to generate individualized targets for neuro-navigated, intermittent theta burst stimulation (iTBS) in order to modulate brain activity in a region centrally engaged in social information processing.

**Methods:**

In this single-blind, randomized, between-subject neuroimaging-guided brain stimulation study we plan to recruit 52 participants with prediagnosed ASD and 52 controls without ASD aged between 18 and 65 years. Participants will be classified into two groups and will randomly receive one session of either verum- or sham-iTBS. Effects will be assessed by using well-established experimental tasks that interrogate social behaviour, but also use computational modelling to investigate brain stimulation effects at this level.

**Discussion:**

This study aims to use personalized, non-invasive brain stimulation to alter social information processing in adults with and without high-functioning ASD, which has not been studied before with a similar protocol or a sample size of this magnitude. By doing so in combination with behavioural and computational tasks, this study has the potential to provide new mechanistic insights into the workings of the social brain.

**Trial registration:**

German Clinical Trial Register, DRKS-ID: DRKS00028819. Registered 14 June 2022.

## Introduction

### Background and rationale

Autism spectrum disorder (ASD) is a neurodevelopmental disorder with an estimated lifetime prevalence of at least 1% and an onset in early childhood [1, 2]. ASD is characterized by qualitative impairments of social interaction and communication as well as stereotyped, repetitive behaviours and sensory abnormalities [1, 2]. A cardinal feature of ASD are deficits in social cognition, including impaired mentalizing [3]. Mentalizing or Theory of Mind (ToM) is the ability to interpret and predict behaviour by ascribing mental states to oneself and others [4]. Mentalizing deficits make it difficult for ASD individuals to quickly grasp others’ beliefs, intentions and emotional states and to adapt their behaviour accordingly during social interaction [4].

Experimental evidence from genetic linkage studies, animal models, functional neuroimaging and non-invasive brain stimulation (NIBS) has shown that ASD individuals show an aberrant cortical plasticity and activity profiles [5–9]. Here, dysfunction of the ‘neurocognitive pathway of mentalizing’ has been demonstrated [10–13]. Based on the insight from previous studies that brain activations differences in the mentalizing pathway might be more closely related to behavioural difficulties and social impairments in autism, we decided to use functional neuroimaging to generate personalized stimulation targets for TMS and to focus on rTPJ as it represents a key node of the mentalizing pathway [14, 15]. It has been proposed that a core feature of ASD without intellectual impairment are individual alterations in functional connectivity patterns. A correlation between the extent of the aberrant connectivity and behavioural symptoms in ASD has also been found [16]. Functional magnetic resonance imaging (fMRI) studies have shown abnormalities in activation and connectivity profiles of the right temporo-parietal junction in autism, supporting its role in situation-awareness and social perception [13, 17]. Moreover, impaired social cognitive abilities and alexithymia have been shown to correspond to reduced connectivity between the dorsomedial prefrontal cortex (dmPFC) and the TPJ [18]. In addition, hypoactivation of the rTPJ during mentalizing tasks has been found to correspond to the severity of autistic symptoms and social impairments [18, 19].

To date there is no causal therapy for ASD and treatment typically consists of psycho-educational approaches and pharmacological interventions for comorbid psychiatric disorders [2]. In the absence of causal treatment, it appears crucial to explore the underlying neurobiology and investigate interventions that may have an impact on brain function in ASD. Here, one important avenue of research is targeting the neurocognitive pathway of mentalizing by means of NIBS in order to facilitate ASD individuals´ inferences regarding other peoples’ mental states [20]. While previous research has provided proof-of-principle evidence that repetitive transcranial magnetic stimulation (rTMS) of the mentalizing pathway can modulate social abilities in patients with ASD, the results of NIBS studies with ASD individuals have not been consistent [6, 21–29]. Still, the majority of the few existing studies investigated the effects of rTMS over the dorsolater prefrontal cortex (dlPFC) attempting to induce NIBS effects on executive functions, but showed no improvement in social deficits [21–25]. Only few TMS studies in small cohorts have targeted other regions of the mentalizing pathway such as the DMPFC or the medial prefrontal cortex (mPFC) [10, 25]. For example, Enticott et al. examined adults with ASD without intellectual impairment with deep rTMS over the DMPFC [28]. Participants showed a reduction in social relating impairment, while no significant effects for the experimental tasks were observed [30]. Avirame et al. reported in a case report that two ASD individuals, who were treated with deep TMS over the mPFC showed improvement in sociability and communication skills [31].

The TPJ, a hub of the ‘neurocognitive pathway of mentalizing’, is a region that has been shown to be under-active in autism and is also functionally coupled with the dmPFC [10, 13, 32, 33]. Besides, the TPJ is due to its location close to the midline structures well accessible for applying TMS. Consequently the TPJ could function as an important and accessible entry point of the neural network that is relevant for processing social cues.

Previous research using NIBS for treatment of depression has repeatedly demonstrated that variation of the stimulation site drastically affects outcome, which makes precision tools indispensable to reduce error and increase response rates [34, 35]. NIBS depression studies discuss currently the benefits of using functional neuroimaging to define the stimulation targets in such a way that relevant networks can be modulated at the individual level [36]. Moreover, brain activation levels vary more strongly between subjects, whereas structural landmarks (i.e. gyri and sulci) do not show this variability [37]. Following this rationale, we implement fMRI-guided TMS in our study. Besides, a newer form of rTMS protocol known as intermittent theta-burst-stimulation (iTBS) has been shown to produce at least similar, if not greater, stimulation effects than traditional rTMS. This protocol has a shorter stimulation duration than conventional rTMS and produces electrophysiological and behavioural changes that outlast the period of stimulation by more than 60 min [38]. Combining this stimulation protocol with fMRI-guided target selection and stereotactic neuronavigation, which allows to track the target in the individual participant, results in a state of the art precise stimulation of the defined target [34, 35].

### Objectives

To optimally probe the effects of rTMS in the rTPJ in ASD, we will combine the afore mentioned state-of-the-art stimulation techniques, including individualized fMRI-guided targets, time-efficient intermittent theta-burst-stimulation (iTBS) and a stereotactic neuronavigation.

In our study on the basis of the current literature the neurocognitive pathway of mentalizing will be localized using a well-established video-based fMRI task, which contrasts brain activation during scenes with socially engaging vis-a-vis socially neutral content [39]. This has been shown to consistently localize activation maxima in regions involved in social cognition including the dmPFC and TPJ on the single-subject level [39]. In a second session, each participant’s activation maximum in the rTPJ will be used as a stimulation target to conduct a neuronavigated iTBS intervention with the aim of modulating social information processing. The effects of the stimulation will be measured by using a test battery encompassing social cognitive as well as cognitive control tasks.

We hypothesize that adults with ASD without intellectual impairment will show an iTBS-induced improvement in social information processing, measured by an established probabilistic learning task that requires the integration of both social and non-social information [40]. In particular, participants with ASD without intellectual impairment belonging to the treatment group will show an increased ability to integrate social information into their decision-making process compared to the control group. We hypothesize that these effects are not due to changes or baseline differences in executive functioning.

Secondary analyses will investigate group differences in executive function tests as well as brain activation and connectivity in the ASD and control group.

## Methods

### Study design and ethics approval

Our study is a single-blind, between-subject, sham-controlled, neuroimaging-guided brain stimulation study, exploring the effects of non-invasive brain stimulation on social cognition and executive function in individuals with ASD without intellectual impairment in comparison to control participants without ASD. Participants will be randomly assigned using block randomization to one of the two stimulation conditions (verum vs. sham) in the beginning of the study. Participant recruitment, neuroimaging, brain stimulation and data collection will be carried out at the Department of General Psychiatry 2 at the LVR-Klinikum Duesseldorf in collaboration with the Institute of Systems Neuroscience, University Hospital Duesseldorf, Germany. Participants will be recruited via advertising flyers, online advertisements and social media. Additionally, participants with ASD will be recruited via the Clinic for Disorders of Social Interaction at the Department of General Psychiatry 2 at the LVR-Klinikum Düsseldorf. This study has been approved by the Ethics Committee of the Medical Faculty Duesseldorf of the Heinrich-Heine-University (ethics approval number: 2021 − 1440), conforms to the requirements of the World Medical Association´s Declaration of Helsinki and is registered at the German Clinical Trial Register (DRKS-ID: DRKS00028819). Written informed consent will be taken by all participants, before entering the study and before any assessments or intervention related to the study are undertaken. Participants will be free to withdraw at any time. If a participant has a legal guardian, informed consent will also be obtained from this person.

### Sample size calculation

A power analysis with an estimated effect size of 0.3, a power of 0.8 and an alpha error of 0.05 was used to determine the required sample size for this study using G* Power [41]. Based on this analysis 90 participants are required. In order to divide the participants in four groups a total sample of 92 participants is required. Taking possible drop-outs as well as loss of data due to technical problems into account we have decided to include 52 participants in each arm of the study, i.e. 52 participants with ASD and 52 participants without ASD. Only little prior evidence exists in the field of neuronavigated TMS in adults with ASD, so that no prior studies could serve as a basis for the power analysis. Since this is a proof-of-concept study we opted for a small effect size, so that even smaller effects of the intervention can be detected.

### Inclusion/exclusion criteria

Participants with an already confirmed ASD diagnosis will be included if they: (1) are 18 to 65 years of age, (2) have not a contraindication for an MRI scan, (3) have an AQ-score equal to or greater than 28, (4) have an IQ greater than 80 and (5) have a BDI II score less than 20. Healthy controls with no history of psychiatric disorders and no psychiatric symptoms as assessed by an experienced consultant psychiatrist during the screening interview will be included if they: (1) are 18 to 65 years old, (2) have not a contraindication for an MRI scan, (3) have an AQ-score equal to or less than 27, (4) have an IQ greater than 80 and (5) have a BDI II score less than 20.

General exclusion criteria will be: (1) known epilepsy, (2) intracranial metallic foreign bodies, intracranial implants and big head tattoos, (3) significant brain malformations, brain tumors, cerebrovascular events (strokes), traumatic brain injuries, neurodegenerative diseases and brain surgeries, (4) implanted cardiac pacemaker, (5) severe illness, (6) tinnitus, (7) glaucoma, (8) pregnancy, (9) medication affecting the seizure threshold, (10) claustrophobia, 11) current or previous treatment with Deep Brain Stimulation or Vagus nerve Stimulation, 12) heavy alcohol consumption (> 60 g alcohol per day) or substance abuse, 13) limited knowledge of German language.

### Experimental procedure

**Fig. 1:**
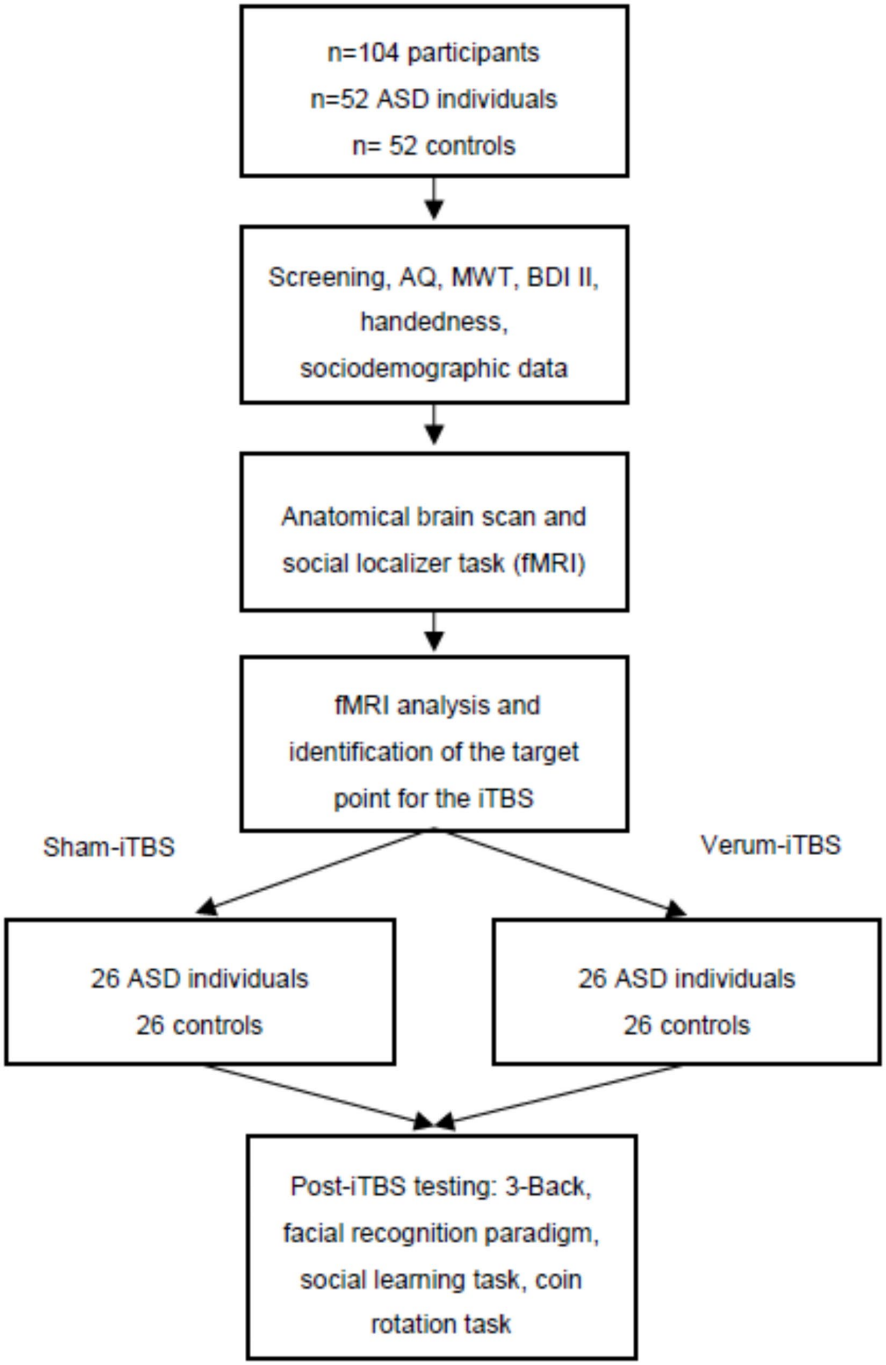
Study flow diagram

An overview of the study plan is depicted in Fig. 1. At the beginning, all participants will undergo a screening session conducted by an experienced psychiatrist so that inclusion/exclusion criteria can be assessed and necessary information can be reviewed. Afterwards informed consent will be obtained and as a part of the screening the medical history will be taken, the Autism Spectrum Quotient (AQ), the Intelligence Quotient (IQ) measured using the multiple-choice vocabulary intelligence test MWT, the Beck Depression Inventory (BDI II) score, the Edinburgh handedness inventory score, as well as sociodemographic data will be collected [42–45]. The data from the screening session will be used in secondary analysis. Participants will be randomly assigned to one of the two stimulation conditions, i.e. verum-iTBS or sham-iTBS. The entire sample will therefore include four groups (ASD individuals receiving verum-iTBS, ASD individuals receiving sham-iTBS, controls receiving verum-iTBS and controls receiving sham-iTBS). All participants will receive an anatomical MRI scan, including a T1-weighted sequence as well as an fMRI scan. During the fMRI scan a well-established video-based social perception task (social localizer) will be administered [39]. In this paradigm audiovisual movie clips displaying pre-selected social clues are presented in order to detect the brain networks subserving perception. Based on this localizer, activation maxima in rTPJ will be extracted at the single-subject level, which will be then used as personalized targets for the neuronavigated iTBS session. Coil positions will be navigated using a frameless stereotaxic neuronavigation system (Localite GmbH). This allows to co-register T1 images of the participants with their anatomical landmarks, enhancing the precision of the stimulation over the fMRI-derived activation peaks of the rTPJ. For the iTBS the following protocol will be used: 600 pulses will be given in 1,84 s trains repeated every 10 s for a total of 189 s at an intensity of 70% of the motor threshold. Stimulations will be performed with a PowerMAG Research 100 stimulator (MAG and More GmbH) and a PMD70-aCool coil (verum-iTBS) as well as a PMD70-aCool-Sham coil (sham-iTBS). Directly after stimulation, the following tasks will be administered in the following order: (1) probabilistic learning task, (2) a facial recognition paradigm, (3) coin rotation task and (4) 3-back task [40, 46–48]. The estimated time required to complete these tasks is approximately 35–40 min. The time of completion of the TMS, as well as the start times of each task, will be recorded. There will be a maximum of 5 min between the completion of the TMS and the start of the social probabilistic learning task. As a result, all tasks will be completed within approximately 40–45 min after the completion of the TMS.

The social learning task, which will be used to measure changes in social cognition, is the only task, which will be administered before and after brain stimulation.

### Outcome measures

(a) In the facial recognition paradigm, the facial affect recognition ability of participants will be assessed using 28 digitally reworked photographs of faces. The photographs show the six basic emotions (happiness, fear, anger, surprise, disgust, sadness and neutral) and have been selected from Ekman and Friesen’s “Pictures of Facial Affect” [47]. Participants will have to choose the emotion that fits best to the shown photograph. For the subsequent analysis, the number of correct responses will be counted. We hypothesize that ASD individuals receiving verum-iTBS will demonstrate better results of facial affect recognition ability compared to ASD individuals receiving sham-iTBS.

(b) The social probabilistic learning task will be administered before and after iTBS [40]. In this task, participants have to integrate social and non-social information to learn which one of two cards leads to a reward. The winning probability of each card will change several times during the experiment, as will the helpfulness of the social cue. The participants will not be explicitly told to use social information. When selecting the winning card on a given trial, the number of points depicted on the card can be won. For the analysis, the main behavioural outcomes are the number of correct responses, the total score accumulated and the number of trials where participants considered social information. In addition, a hierarchical Gaussian filter model will be used to mathematically model how beliefs about the helpfulness of the social cues are learned and integrated with non-social beliefs about the probability of reward for each stimulus. Previous studies using dynamic learning tasks in conjunction with computational modeling have shown that high in autistic trait individuals find it difficult to integrate social and non-social cues and to form predictions about volatile environments [49, 50]. We hypothesize that ASD individuals receiving verum-iTBS compared to control treatment will demonstrate an increase in the number of correct responses and in the proportion of trial where they follow social cues. With computational modelling, we aim to examine the underlying cognitive mechanisms that bring about these behavioral outcomes. We hypothesize that iTBS will improve the ability to integrate social and non-social information, as measured by a social weighting parameter, while not having an effect on how participants update beliefs in the non-social domain.

(c) The 3-back task will be used as a control task to measure a domain of the executive functions by testing participants’ working memory and memory capacity [46]. Participants will be presented with a series of alternating stimuli (numbers) changing in random sequence, eventually repeating and will be asked to report when a stimulus matches another stimulus presented three trials back (*N* = 3). The number of correct responses and reaction time will be counted. No improvement in participants´ executive function ability after neuronavigated iTBS is anticipated.

(d) The coin rotation task, also a control task, will be used to access participants´ fine motor skills [48]. Participants will be asked to take a coin between their thumb, forefinger and middle finger and spin it between these fingers as quickly as possible without dropping it for 15 s. Three attempts will be allowed but only the second and third will be counted. The number of 180^o^ coin rotations in 10 s will be counted. The performance will be recorded with a camera and further analyzed offline using slow motion. We hypothesize that ASD participants will not demonstrate improvement in their motor skills after iTBS.

Table 1 offers an overview of the key instruments that will be used in the study.

**Table 1:** Overview of instruments

Instruments	Measured construct
Autism Spectrum Quotient	Autistic traits
Intelligence Quotient/MWT	Mental ability
Beck Depression Inventory	Depressive symptomatology
Social localizer	Brain connectivity
N-back task	Working memory
Facial recognition paradigm	Affect recognition
Social task	Probabilistic learning
Coin rotation task	Motor skills

### Analysis plan

#### Demographic data and behavioral tasks analysis

All behavioral data will be analyzed using the statistical software IBM SPSS Statistics. For the analysis a two-way factorial ANOVA will be used. More precisely for the 3-back task response accuracy will be recorded and two-way ANOVAs will be used for comparisons between groups. For the facial recognition paradigm, the number of correct responses will be recorded and two-way factorial ANOVAs will be used for between groups comparisons. For the coin rotation task, the number of 180° coin rotations in 10 s will be recorded and two-way factorial ANOVAs will be used for comparisons between groups.

The probabilistic learning task will be analyzed using the Hierarchical Gaussian Filter (HGF) model to infer participants’ individual belief trajectories concerning the cards and the social cues. Model comparisons will be carried out by means of Bayesian Model Selection. Group-specific differences in model parameters will be evaluated by means of mixed ANOVAs with schedule stability as a within-subject factor, information type as the within participant factor (social vs. card). Groups and brain stimulation will be the between subject factors.

#### fMRI acquisition and analysis

MR imaging will be carried out with a Siemens MAGNETOM Prisma 3.0 Tesla scanner using a 64-channel receiving coil including functional (EPI sequence, TR: 800ms, TE: 37ms, Flip angle: 52°, Multiband acceleration factor: 8, marix: 104 × 104, in-plane resolution: 2 mm x 2 mm. slice thickness: 2 mm, slice gap: 2 mm, slice count: 72, slice orientation: obligue) and T1 (MPRAGE sequence, TR: 2500ms, TE: 2.22ms, TI: 1000ms. FIlip angle: 8°, marix:300 × 320, resolution: 0.8 mm isotropic, slice count: 208, slice orientation: sagittal) anatomical scans. Stimuli will be controlled and synchronized with the scanner pulses with Presentation^®^ (Neurobehavioral Systems Inc., Berkeley, CA) software. Visual stimulation will be delivered to the participants from an 40” MRI compatible flatscreen display (InroomViewingDevice, NordicNeuroLab AS, Bergen, Norway) through a mirror attached to the head coil. Auditory stimulation is delivered via Sensimetrics S14 insert earphones (Sensimetrics Corporation, Malden, Massachusetts, USA) driven by a t.amp S-100 MK II (Thomann GmbH, Burgebrach, Germany) analog power amplifier. Functional data will be preprocessed and analyzed with SPM software. Preprocessing will include removal of the first four volumes, realignment and unwarping the functional data and co-registering it to the structural image. Tissue segmentation will be performed on the anatomical images. Finally, a 6-mm spatial smoothing full-width-at-half-maximum Gaussian kernel will be applied to the functional images to reduce the effects of uncorrelated noise. Analyses will be performed in the subject space to avoid biasing the activation loci by spatial normalization. For each participant, we will use a general linear model (GLM) to assess regional effects of the eight social and six non-social features on the BOLD signal that were collected during a previous study [39]. The model consists a column of events and associated parametric modulators for the social and non-social features for each volume of the fMRI data. Individual motion parameters will be included as nuisance covariates. To detect the region in the rTPJ that is maximally selective for social information, a t-contrast will be calculated contrasting the mean responses to the social vs. non-social categories. After the analysis, the ensuing statistical T-maps as well as the structural T1 image will be transferred to the stereotactic neuronavigation system in order to place the TMS coil over the portion of the rTPJ featuring the highest engagement in social perception in each participant.

## Discussion

This paper presents a single-blind randomized, personalized TMS study protocol investigating whether neuronavigated iTBS can modulate social cognition in adults with ASD without intellectual impairment. With this study, we aim to provide new mechanistic insights into the neurocognitive processes that underlie ASD. We do so by combining neuroimaging-guided brain stimulation with a behavioral test battery that also includes an established computational modeling task, which has been successfully used to formally describe the cognitive processes that underlie social and non-social information processing in different diagnostic groups. Here, it was shown that autistic traits are, indeed, associated with performance differences. Interestingly, it was shown that these trait-related performance differences are not explained by an inability to process the social stimuli, but rather by the extent to which participants consider social information to infer the non-social cue. We hypothesize that combining personalized brain stimulation of a brain region that is known to play a central role in social inference processes will be effective in modulating these processes.

To the best of our knowledge, no prior studies have undertaken an investigation of this topic with a sample size of this magnitude and similar tasks.

One challenge of this study will be to recruit a relatively large number of participants with ASD without intellectual impairment that are willing to undergo iTBS. Adults with ASD may also exhibit reluctance to engage in the study due to concerns related to stress caused by social interaction with the researchers, however carrying out the study in a hospital featuring a sizeable outpatient clinic specialized in ASD will hopefully help during the recruitment process.

An important strength of this study will be its relatively large sample size. As already mentioned, the few existing TMS studies in adults with autism have had relatively small sample sizes [29]. Our target of 104 participants will ensure statistical power to test our hypothesis. Another strength of the current study is the use of neuronavigation to localize the personalized target, which ensures accurate and consistent targeting of the region of interest, thus minimalizing variability and increasing validity of our findings. Furthermore, the utilization of iTBS is a strength, as it offers a more time-efficient and potentially more effective stimulation paradigm [38]. A limitation of this study could be employing only a single session of TMS, as multiple sessions could yield cumulative effects and better measurable results. Another limitation could be the single-blind study design. However, the primary endpoint tasks are highly standardized and the entire evaluation process is computerized, thereby minimizing any effects arising from the lack of blinding.

If this study succeeds in demonstrating that personalized TMS can modulate social cognition in adults with ASD without intellectual impairment, this may serve as an important stepping stone towards studies with larger sample sizes, more TMS sessions and maybe different TMS protocols.

## Data Availability

The datasets generated and/or analyzed during this study will not be publicly available, but will be available from the corresponding author upon reasonable request.

## Abbreviations

ASD: Autism spectrum disorder
ToM: Theory of mind
NIBS: Non-invasive brain stimulation
rTPJ: Right temporo-parietal junction
dmPFC: Dorsomedial prefrontal cortex
dlPFC: Dorsolateral prefrontal cortex
mPFC: Medial prefrontal cortex
fMRI: Functional magnetic resonance imaging
iTBS: Intermittent theta-burst-stimulation
AQ: Autism spectrum quotient
IQ: Intelligence quotient
BDI: II Beck depression inventory
MWT: Multiple-choice vocabulary intelligence test
GLM: General linear model
HGF: Hierarchical gaussian filter
